# Supplementary material for: SerpinB3/4 Expression Is Associated with Poor Prognosis in Patients with Cholangiocarcinoma
Source: Cancers (Basel). 2024 Jan 3;16(1):225. doi: 10.3390/cancers16010225 (PMC10778206; doi:10.3390/cancers16010225)
Supplement: Supplementary file 1 [file cancers-16-00225-s001.zip › cancers-2769609-supplementary.pdf]

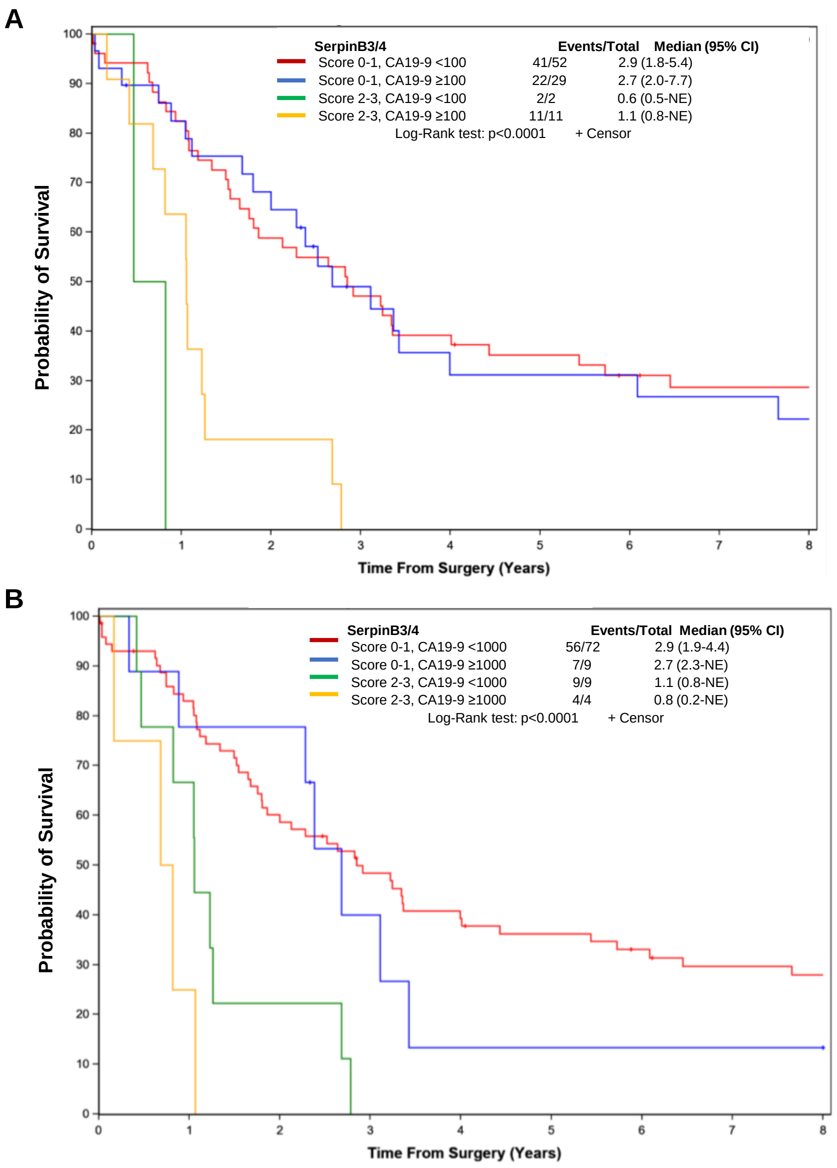

Suppl. Figure S1. Overall Survival in CCA cases divided according to the SerpinB3/B4 score and different serum levels of CA19-9. Overall survival was assesses in 123 CCA patients, divided according to the SerpinB3/B4 score, determined by immunohistochemistry (high SB3 = score 2-3, low SB3 = score 0-1) and different serum levels of CA19-9. In **Panel A** the CA19-9 cut-off of 100 U/mL and in **Panel B** the CA19-9 cut-off of 1000 U/mL were considered. Survival Curves were estimated using Kaplan- Meier method and the differences between curves was assessed by long-rank test.
